# Nanogels as nanocarriers for drug delivery: A review

**DOI:** 10.5599/admet.724

**Published:** 2019-12-21

**Authors:** Rahul Rajput, Jitendra Narkhede, Jitendra Naik

**Affiliations:** University Institute of Chemical Technology, KBC North Maharashtra University, Jalgaon, 425001, India

**Keywords:** Drug, Polymers, Nanogels, Drug delivery, Applications

## Abstract

Nanogels are submicron-size aqueous dispersions of water-swollen particles, composed of nano-sized three-dimensional highly cross-linked networks of hydrophilic polymers. An active pharmaceutical agent or therapeutic agent with high or low molecular weight can be easily encapsulated into nanogels that can be delivered to the site of action via various routes, including oral, pulmonary, nasal, parenteral and intraocular routes, among others. Therapeutic agents encapsulated into nanogels improve the therapeutic activity in the biological environment. The application of different nanogels in drug delivery and recent clinical trial studies has been described concisely in this review.

## Introduction

Nanogels are commonly used in sensing, diagnostics, and bioengineering, but they are also often used in drug delivery [1, 2]. Nanogels have benefits over conventional and macro-sized delivery systems because of their higher drug loading capacity, high stability, and improved contact time with the skin surface, which makes it more convenient as a transdermal drug delivery system. Water-soluble non-ionic polymers like hydroxylpropyl methylcellulose as well as ethylcellulose are commonly used to stabilise nanogel dispersions [3-7]. Phase separation of drug-loaded nanogels could occur due to interactions (electrostatic, hydrophobic, van der Waals) between the polymeric matrix and the active agent, which could be prevented by dispersing hydrophilic polymers. The dispersed hydrophilic polymer becomes exposed to the skin surface by forming a protective layer around the nanogel, allowing drug particles to remain dispersed in the gel matrix [8-10]. Modified natural biopolymers possess a high degree of functional groups with additional functional cross-linkers used for the formulation of biopolymer-based nanogels. Innovative techniques such as photopolymerisation, chemical cross-linking, click chemistry-based cross-linking etc., are used to derive the self-assembly and cross-linking of hydrophilic block copolymers. Between internal and external layers of nanogels, block polymers permit the control of drug release from a polymer matrix [11-13]. For target-specific or cell-specific drug delivery, nanogels are modified with ligands to permit receptor-mediated drug release at the site of action [14, 15]. Drug- or biologically-loaded nanogels cross biological barriers and release the therapeutic agent inside cells [16-18]. In recent years, nanogels were effectively utilised in the field of biotechnology to deal with genetics, enzyme immobilisation and protein synthesis, thus providing an efficient tool to cater for novel therapeutic systems in medicine (Table 1). A novel core-shell magnetic nanogel was prepared using poly(acrylamide) for cancer therapy. Pluronic poly(ethyleneimine) was used to prepare a thermoresponsive nanogel for the transdermal delivery of an active agent. Nanogel-based drug delivery formulations increase the effectiveness and safety of certain anti-cancer drugs as well as many other drugs due to their chemical composition, which has been confirmed by *in vivo* studies in animal models. Nanogels are a favourable and innovative drug delivery system that can play a vital role by addressing the problems associated with old and modern therapeutics such as nonspecific effects and poor stability [19-23].

## Methods used for the preparation of nanogels

- Preparation of nanogel using polymeric precursors
- Synthesis of nanogel network by heterogeneous polymerisation of monomers

Polymers and monomers having different nanoscopic structures formed by amphiphilic copolymers are used for the preparation of nanogels. Amphiphilic copolymers are able to self-assemble in solution, hence they form nanogels [24, 25].

### I) Preparation of nanogels from polymeric precursors

#### Disulphide-based cross-linking

Disulphide linkages were prepared by inverse mini-emulsion atom transfer radical polymerisation (ATRP). In this process, water-soluble monomer oligo(ethylene glycol) monomethyl ether methacrylate (OEOMA) with different molecular weights were cross-linked in the ATRP reaction with the disulphide-functionalised cross-linker. The nanogels formed are considered to have a uniformly cross-linked network, which is supposed to improve aqueous solubility and control the release of encapsulated agents; the nanogels are shown to be biodegradable into water-soluble polymers in the presence of a biocompatible glutathione tripeptide which is commonly found in cells. The biodegradation of nanogels could trigger the release of drugs from the nanogels. Amphiphilic random copolymers are used to prepare a nanogel system by self-cross-linking [26, 27]. A nanosized product in aqueous solution is formed by hydrophilic poly(ethylene glycol) and pyridyl disulphide. Thiol disulfide exchange reaction is depending primarily on the concentration of thiol exchangers like dithiothreitol (DTT). The size of the nanogel would be reduced by using cross-linking monomer or polymer chains. A lower critical solution temperature (LCST) of polymers also affects the size of the nanogel. Lipoic acid-encapsulated dextran was prepared by thiol-exchange using the same method. A catalytic amount of DTT freely cross-linked with doxorubicin was synthesised from the assembly of the polymer [28, 29].

**Figure 1.**
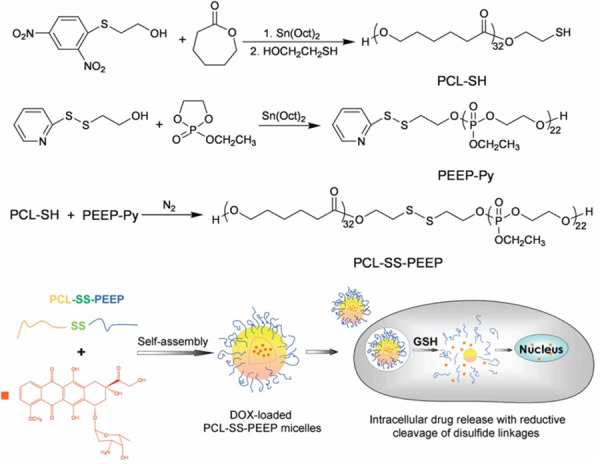
Synthesis pathway of the disulphide-linked PCL-SS-PEEP and schematic illustration of intracellular drug release (reprinted with the permission from [30]. Copyright (2016) American Chemical Society).

Poly(ε-caprolactone) (PCL) and hydrophilic poly(ethylethylenephosphate) (PEEP) were used as drug carriers for the development of a micellar nanoparticle system for intracellular drug release which is triggered by glutathione in tumour cells. Tang *et al.* synthesised a disulphide-linked di-block copolymer of PCL and PEEP (PCL-SS-PEEP), which forms biocompatible micelles in aqueous solution and detaches the shell material under glutathione stimulus, resulting in rapid drug release with the destruction of the micellar structure shown in Figure 1 [30].

Amine group is more common in amine-based cross-linked nanogel development because the amine group has higher reactivity against carboxylic acids, isocyanates, and iodides. Cross-linked knedel-resembling structures using amine cross-linkers were prepared by the Wooley group. Hydrophilic, amphiphilic di-block copolymers were prepared by reversible addition fragmentation chain transfer polymerisation. Amidation of carboxyl group-containing self-assembled block copolymers with a diamine cross-linking agent leads to the cross-linking of micellar assemblies; the remaining carboxylic group was altered for orthogonal surface modifications in the form of other functional moieties to form a cross-linked nanogel. Furthermore, reaction with isocyanate carriers is an alternative cross-linking approach to prepare nanogels. pH-responsive cross-linked micelles were prepared by the addition of 1,8-diaminooctane to a micellar combination of 3-isopropenyl-α,α-dimethyl benzyl isocyanate bearing copolymers [31, 32].

**Figure 2.**
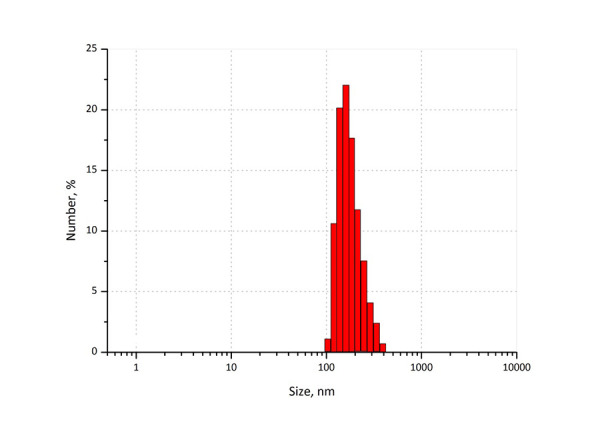
Average particle size of luli-conazole loaded poly(acrylic acid) nanogel

We prepared poly(acrylic acid) (PAA) and sodium carboxymethylcellulose (NaCMC)-based luliconazole loaded nanogels. Luliconazole was encapsulated in PAA and Na-CMC by free radical polymerisation. Luliconazole is an azole antifungal that works by preventing the growth of the fungus [33] and is used to treat skin infections such as athlete's foot, jock itch, and ringworm. Figure 2 shows particle size analysis for the optimised nanogel containing luliconazole. The average particle size of nanogel was 259 nm at the count rate of 360 kcps with polydispersity index (PDI) of 0.2 showed narrow particle size distribution.

**Figure 3.**
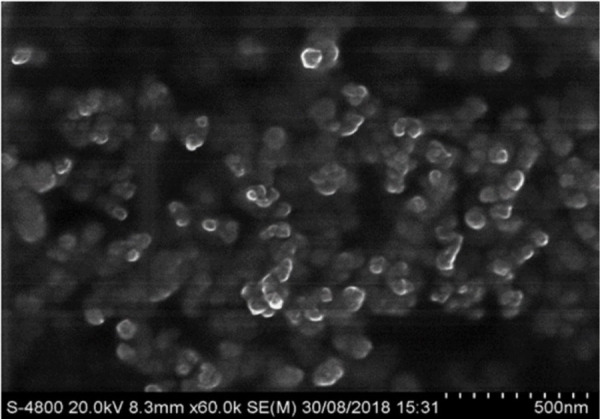
FESEM image of luliconazole loaded poly(acrylic acid) nanogel

The formation of nanogel (NaCMC-g-PAA) from NaCMC and acrylic acid/sodium acrylate in the presence of N,N’-methylene bisacrylamide (MBA) was carried out using potassium persulphate as a free radical initiator [34, 35]. Figure 3 shows the FESEM micrograph of the optimised nanogel formulation. From the figure, it can be observed that the formed nanogel is spherical in nature.

#### Click chemistry-based cross-linking

This method is discovered by Wooley and Hawker group for the synthesis of nanogels [36]. In this method, alkynyl groups were restrained to the corona of assembled micelles prepared from amphiphilic di-block copolymers of poly(acrylic acid)-b-polystyrene via the amidation of acrylic acid groups. Azido dendrimers and micelles are generally prepared by click reactions. Covalently cross-linked micelles are entrapped into the nanogel assemblies. Cross-linked polyion nanogel micelles were prepared by Liu *et al.* using a click chemistry approach. Core cross-linked polyion complex micelles had thermosensitive coronas with high stability against salt and pH [36, 37].

Thermoresponsive poly(N-isopropylacrylamide) (PNIPAM) represents an attractive candidate to introduce physical cross-linking via the association of hydrophobic domains because it has a gelling temperature below body temperature and good biocompatibility. PNIPAM is a non-biodegradable, thermo-reversible hyaluronan-poly (N-isopropylacrylamide) (HA-PNIPAM) hydrogel with a well-defined molecular architecture and properties; this hydrogel can be synthesised through reversible addition fragmentation chain transfer (RAFT) polymerisation and “click” chemistry polymerisation method [38].

**Figure 4.**
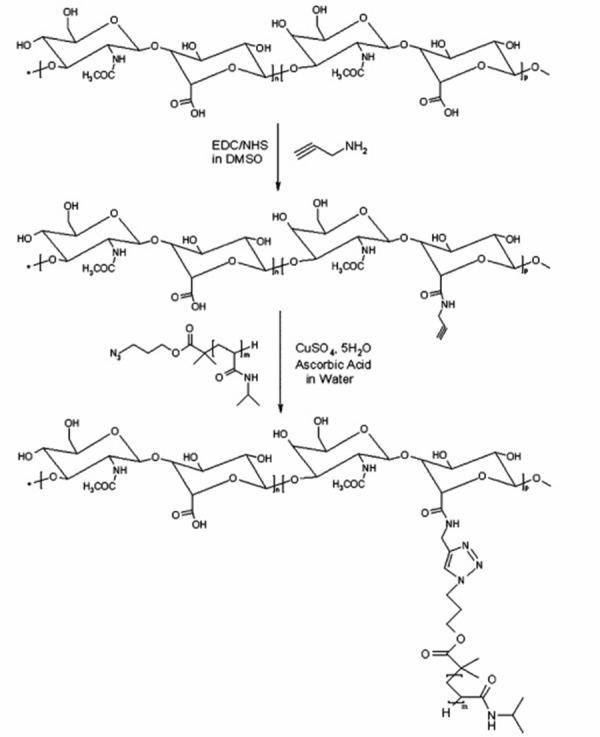
N-(3-dimethylaminopropyl)-N-ethylcarbo-diimide hydrochloride (EDC), N-hydroxy-succin-imide (NHS) synthesis of hyaluronanpropargyl-amide (hapa), followed by copper-catalysed azide-alkyne cycloaddition of hapa with azido-terminated poly(N-isopropylacrylamide) N3-PNIPAM (reprinted with the permission from [38]. Copyright (2010) American Chemical Society).

The effect of PNIPAM grafting length and density on HA-PNIPAM properties was evaluated by methods relevant for cell therapy. It was found that the reversibility of the PNIPAM gelling process was improved in the presence of HA. The efficiency of the “click” reaction facilitates the control of the DS of PNIPAM chains. RAFT polymerisation allows the preparation of PNIPAM of controlled molecular weight and low PDI. This control of the critical parameters of PNIPAM molecular weight and grafting density allowed the gel to be optimised for regenerative medicinal applications. The two synthetic steps of HA-PNIPAM are shown in Figure 4. The EDC/NHS mediated coupling of PPA to carboxylic acid groups on the hyaluronan salt and the copper-catalysed azide-alkyne cycloaddition of the N3-PNIPAM to the hyaluronan-propargylamide [38-40].

#### Photo-induced cross-linking

**Figure 5.**
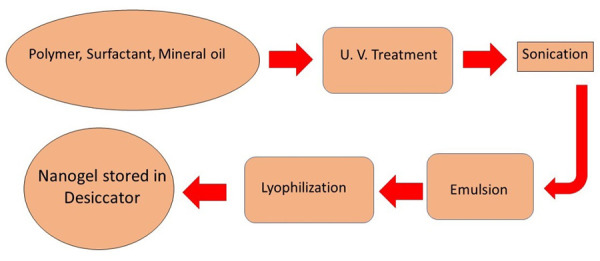
Scheme for UV photopolymerisation

This is an alternative method for nanogel preparations. In this method, the polymer chain is stabilised using cross-linking and is functionalised with dimerisable or polymerisable units. Double hydrophilic block copolymers can be encapsulated into the coumarin dimer. Coumarin dimers, when cured with UV light >310 nm, are assembled into micelles and then photo-cross-linked to form nanogels (Figure 5).

**Figure 6.**
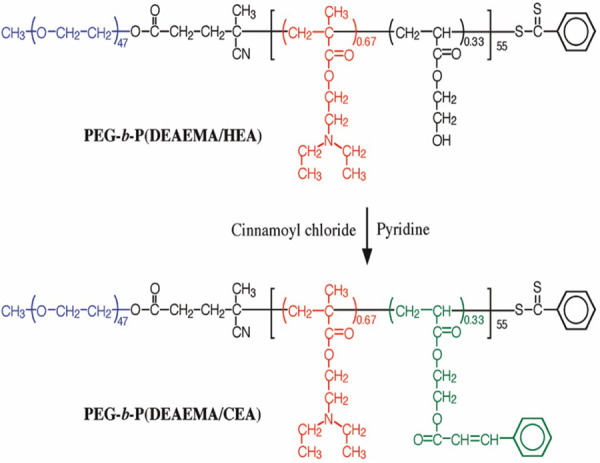
Synthetic route for poly (ethylene glycol)-b-poly(2-(diethylamino) ethyl methacrylate-co-2-cinnamoyloxyethyl acrylate) (reprinted with permission from [45]. Copyright (2009) American Chemical Society).

This nanogel shows interaction between lower critical solution temperature (LCST) and upper critical solution temperature (UCST) behaviours. UCST is the critical temperature above which the contents of a mixture are miscible. The light penetrating capability has been incorporated into dendrimer structures to increase drug release in response to light stimulation. Dendrimers of coumarin act as alternative cross-linkers to control the accessibility of substrate in the nanogel networks. When the solution of coumarin was cured in UV light, the ester groups were confined in the interior assembly of nanogel. Enzymatic degradation of the substrate was very much inhibited. Curing the cross-linked assembly by UV light improves the enzymatic action due to the de-cross-linking of the coumarin dimer, which exposes the substrate to enzymes [41-43]. A stimuli-responsive nanogel prepared in water by a core cross-linking technique using diblock copolymer micelles by photo-cross-linking of the micelle core [44]. The preparation of poly(ethylene glycol)-b-poly(2-(diethylamino) ethyl methacrylate-co-2-cinnamoyloxyethyl acrylate) (PEG-b-P(DEAEMA/CEA)), a pH-responsive block copolymer, by reversible addition-fragmentation chain transfer (RAFT)-controlled radical polymerisation using poly(ethylene glycol)-based chain transfer agent (PEG-CTA). The poly(ethylene glycol) (PEG) block in PEG-b-P(DEAEMA/CEA) is soluble in water, independent of pH, while the solubility of DEAEMA depends on pH (Figure 6) [44, 45].

**Figure 7.**
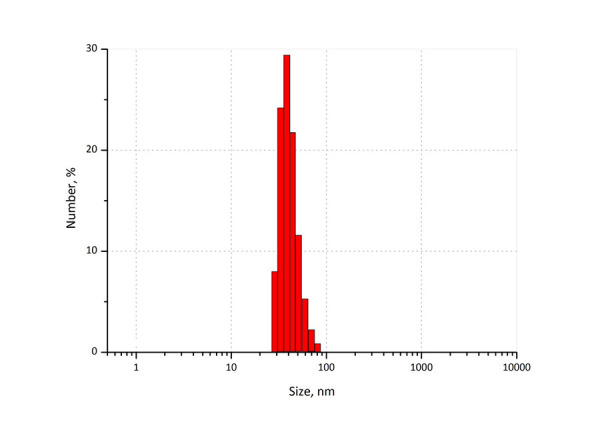
Average particle size of thymol loaded polyacrylamide nanogel prepared by photopolymerisation

**Figure 8.**
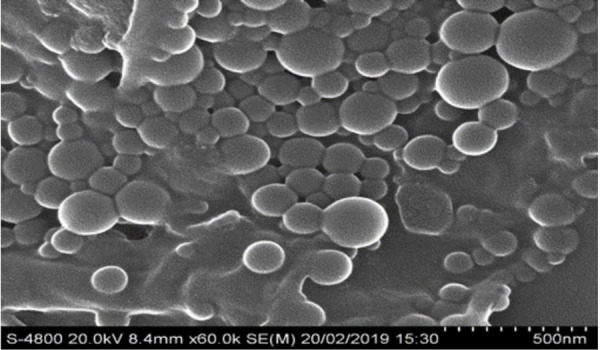
FESEM image of thymol loaded poly(acrylamide) acid nanogel prepared by photopolymerisation

We have formulated poly(acrylamide) (PAA) nanogel using UV polymerisation. PAA gels are mainly polymerised using catalysts such as tetramethylenediamine (TEMED) and ammonium persulphate (APS), which are highly toxic and lead to slow polymerisation, which is time-consuming and takes 45 min to 1 h for lower gel precursor concentrations. Photo-crosslinking with various photoinitiators, such as Irgacure 2959, has been more recently used for the synthesis of PAA hydrogels with a stiffness gradient and used for the quick preparation of large PAA hydrogel arrays for applications such as drug screening. Photo-crosslinking circumvent the use of toxic catalysts and is characteristically much faster than chemical cross-linking method. Final properties of UV-polymerized gels are depending on the UV wavelength, consistency, light intensity, and interaction time [46]. Figure 7 shows the average particle size of thymol loaded PAA nanogel and Figure 8 shows the FESEM micrograph of thymol loaded PAA. It is clear from the figure that the nanogel prepared by UV-polymerisation is spherical in nature.

#### Physical cross-linking

Physically cross-linked gels are also known as pseudo gels which have weaker van der Waals linkages, hydrogen bonding, hydrophobic or electrostatic interactions that are involved in the synthesis of pseudo gels. The physicochemical properties of gels depend on properties of the polymer, temperature, ionic strength, concentration of polymer and the cross-linking agent. Combination of amphiphilic block copolymers and complexation of oppositely charged polymeric chains is used for the formulation of pseudo gels [43, 47].

### II) Synthesis of nanogel networks by heterogeneous polymerisation of monomers

Bi-functional monomers are chemically entrapped into nanogels. Heterogeneous colloidal systems are responsible for the activation of polymerisation. Emulsion polymerisation and ATRP are used for the preparation of biodegradable nanogel. Disulphide-linked bi-functional monomers are used in the stimulation of polymerisation. Protein nanogel hybrids using ATRP in water/oil mini emulsions or inverse mini-emulsion are useful for the entrapment of covalently bonded proteins into nanogel. In the inverse mini-emulsion, a co-initiator was used to initiate the polymerisation of monomers which are firmly dispersed in the system [48, 49].

## Drug release mechanism of nanogel

### pH-responsive mechanism

pH-responsive, nanosized nanogels have received significant attention because of their biological relevance and due to their potential applications in drug delivery systems. Drug release is affected by the different pH values throughout the human body physiological conditions. pH-responsive block copolymer micelles are suitable for controlled delivery applications. In such applications, however, the polymer micelles may experience dilutions below the critical micelle concentration (cmc), leading to dissociation into monomers. In contrast, nanogels with a cross-linked structure are robust at a diluted concentration. Insoluble 3D structures and staying alive at low pH are the main characteristics of methacrylic acid ethyl acrylate. The polymeric chain repulsions begin and lead to the precise release profile in procaine hydrochloride due to the cumulative pH ranges of acidic group ionisation. Suitable pH at the site of action helps with the diffusion of nanogels. pH-responsive monomers play an important role in the preparation of nanogels; these are commonly pH-responsive functional groups that deionise in the polymeric assemblies. A nanogel containing platinum nanoparticles exhibited on and off catalytic activity for shifting reactive oxygen types. In the acidic pH of skin, the protonation of pendant amine of cross-linked poly(2-(N,N-diethylamino) methacrylate) core as well as PEG group in the polymer greatly enhances the solubilisation of drug [43, 50, 51].

### Thermosensitive and volume transition mechanism

Variations in the capacity of nanogels according to temperature are known as the volume phase transition temperature (VPTT). Polymers become quenched and hydrated when the surrounding medium is below the VPTT. A shrunken and hydrated polymer swells and releases the loaded therapeutic agent. Thermo-responsive nanogels rupture in cells and the biological environment when they swell and rise in volume. N-isopropyl acrylamide synthesised nanogels have thermoresponsive properties. These nanogels have important characteristics, such as rapid contraction in gel volume and the efflux of indomethacin due to the maintenance of heat beyond the lower critical solution temperature (LCST). The poly(N-isopropylacrylamide-co-acrylamide)-loaded 5-fluorouracil gel has been tested on rats in *ex vivo* studies. The loading of the therapeutic agent at lower temperatures and the release from nanogels at body temperature makes this suitable for drug delivery. Pluronic acid-modified thermoresponsive poly(ethyleneimine) nanogels were effectively used as gene delivery systems. Thermoresponsive nanogels with PNIPAM have very exciting and promising applications in the biomedical field, such as the treatment of certain cancers through hyperthermia. They can be loaded with an anticancer drug and, at the target location, by moderately increasing the temperature above the LCST, the nanogel can change with volume and the drug release can be increased [52].

### Photoisomerisation and photochemical internalization

Stimulation of photosensitiser-loaded nanogels leads to the synthesis of singlet oxygen and reactive oxygen species which causes oxidation of cellular compartment walls such as endosomal barrier walls; this affects the release of therapeutics into the cytoplasm. An azo dextran nanogel loaded with aspirin showed the e-configuration of the azole group rather than the z-configuration at 365 nm; cis-trans isomerisation of azobenzene by photo-regulation in an azo-dextran nanogel loaded with aspirin as a model drug exhibited that the e-configuration of the azo group leads to a better release profile of the drug than the z-configuration at 365 nm radiation [12, 43, 53].

### Miscellaneous examples

Degradation of disulphide linkages in cross-linked hyaluronic acid nanogels causes the degradation of the nanogel assembly due to the action of reducing agents; in this way, doxorubicin is released by the simple diffusive process. The size of the nanogel increases and the layer by layer release of an active ingredient is possible without a rapid burst of the drug. The release can be sustained by simple diffusion and controlled following initial release mediated by a coating with anionic and cationic polyelectrolytes [54].

## The application of nanogels

Nanogel-based drug delivery formulations improve the effectiveness and safety of anti-cancer drugs, antifungal drugs, and anti-diabetic drugs, due to their physicochemical properties, as well as improving the ease of administration, as confirmed by *in vivo* studies. Nanogels have minimum toxicity to nearby tissues and high healing effects in cancer treatment at the site of action [55].

### Transdermal drug delivery of an antipyretic drug

The nanosized dispersion of aceclofenac was formulated by emulsion-solvent diffusion methods and then incorporated into a Carbopol 940. The formulation showed optimal permeability properties and stability, and achieved a sustained drug release. A nanogel formulation containing diclofenac sodium was prepared by the emulsion-solvent diffusion method and then incorporated into a Carbopol 940 [56].

### Ophthalmic applications

Curcumin-loaded cationic nanostructured lipid carriers (CNLC) were prepared by film-ultrasonic techniques and thermosensitive gelling agents were used to improve pre-ocular retention and the ocular permeation capacity of curcumin. Muscone has maximum drug loading in the hydrogel, and the rheology results showed that the phase transition temperature was 34°C. Blinking of eyes was resisted due to the thixotropy; the recovery time indicated that hydrogel was effective [43, 57].

### Diabetic applications

In diabetic patients, insulin is injected into muscles every day, which is a very painful process. To overcome this problem Lee et al. (2012) developed a chitosan-loaded inhalable deoxycholic acid altered glycol chitosan (DOCA-GC) nanogel. Nanogels are self-assembled due to hydrophobic attractions with deoxycholic acid; these nanogels formed constant hypoglycaemia over a period of 2 days comparatively at the low dose [58].

### Carrier for antifungal agents

In fungal infections, physicians and patients prefer the transdermal route. A fluconazole-chitin nanogel was formulated by using regeneration chemistry and the wet milling method. Chitin nanogels were redeveloped from chitin solution. Fluconazole-chitin has a controlled release pattern which is perfect for the continuous availability of fluconazole over a longer period for effective fungal treatment [59]. The synthesis of a vitamin E nanoemulsion-based nanogel consisting of the high molecular weight active agent amphotericin B has been effectively used for cutaneous fungal infections; the nanogel showed a nearly 4-fold higher skin deposition through porcine ear skin [60].

### Nanogels in diagnostics and imaging

Nanogels have properties like structural flexibility, high water content, fluid-like transport, biodegradability, and biocompatibility. Gadolinium-assembled nanogels were synthesised by the cross-linking of branched polyethyleneimines with gadolinium ions. Inverse microemulsion followed by surface-functionalisation with polyethylene glycol chains was performed to increase the blood circulation time [61].

## Properties of nanogels

### High water content/swellability

Nanogels have rapid swelling and de-swelling properties. Water-soluble nanogels show the benefits of hydrogels with certain advantages that are necessary for their nanoscale size. Like microgels, nanogels can contain and protect drugs and regulate their release by integrating high-affinity functional groups containing polymers [43, 62].

### Softness

The softness of nanogels is a very important parameter in the biomedical field and alters their biodistribution properties. Softness can be adjusted by changing the chemical structure of the nanogel [63].

### Colloidal stability

The surface charge of polymers inhibits the development of aggregates in the bloodstream, along with their associated problems. This can be altered by increasing the zeta potential that results in higher repulsive forces between particles which electrostatically stabilise nanogels. Another method includes the integration of surfactants like polyethylene glycol which produce a steric effect and hydration forces to give a stable nanosuspension [43, 64].

### Biocompatibility and degradability

Natural or synthetic polymers are used to synthesise nanogels. These are biocompatible and biodegradable, thereby preventing their accumulation in the systemic circulation. Chitosan, poly-acrylic acid, methyl cellulose, sodium alginate, and several polysaccharide-based polymers like dextran, pullulan, and cyclodextrin can be used to formulate nanogels. Polysaccharides are typically carbohydrate-based polymers formed of repeating monosaccharide units linked by glycosidic bonds. These polymers are stable, non-toxic, hydrophilic and biodegradable in nature [65].

### Particle size

Nanogels are able to diffuse through the skin, tissues or compromised areas of the endothelium and in some cases through a specific transport system. Some routes of administration face the challenge of crossing the blood-brain barrier (BBB) due to their particle size. So, to overcome this issue, nanogels were developed which have a size in the diameter range from 20-200 nm. Nanogels have smaller sizes, so cross the BBB while inhibiting rapid clearance mechanisms at the same time [43, 66].

## Concluding Remarks

Nanogels are advanced pharmaceutical nanocarriers for pharmaceutical agents as well as therapeutic agents. Nanogel systems could be easily prepared with biomacromolecules with the maximum entrapment ability and stability of the resulting formulation in dispersion. Nanogel systems control pharmaceutically-active compounds with different drug structures. Biopolymers and low molecular mass hydrophobes can also be encapsulated in nanogels. The discovery of a new polymeric system is very important for the development of nanogels. Advanced polymerisation or cross-linking approaches have a promising role in therapies. This is a new approach in the synthesis of nanogel assemblies. Hence, we can expect that these advanced nanocarrier systems will be focused upon in future pharmaceutical developments.

## Figures and Tables

**Table 1.** Applications and types of nanogel in drug delivery [21-23]

Polymer	Type of Nanogel	Uses
Pullulan/folate-pheophorbide	Self-quenching polysaccharide-based	Minimal phototoxicity of pheophorbide
Cross-linked branched network of poly(ethyleneimine) and PEG	Polyplex nanogel	Elevated activity and reduced cytotoxicity of fludarabine
Acetylated chondroitin sulphate	Self-organising nanogel	Doxorubicin loaded
Heparin pluronic nanogel	Self-assembled nanogel	RNA enzyme delivery internalized in cells
Poly(ethyleneimine) nanogels	Size-dependent property nanogel	Suicide gene hTERT –CD-TK delivered for lung cancer
Poly(N-isopropylacrylamide) and chitosan	Thermosensitive magnetically modalised	Hyperthermia cancer treatment and targeted drug delivery
Poly(acrylamide)	Novel core shell magnetic nanogel	Radiopharmaceutical carrier for cancer radiotherapy
Methylacrylic acid and N,N’-methylene-bis-(acrylamide)	Supermagnetic nanogel functionalised with carboxyl group	α-chymotrypsin immobilized on aminated nanogel
Methylacrylic acid and N,N’-methylene-bis-(acrylamide)	Magnetic nanogel hydrophilic polymers	α-chymotrypsin immobilized on carboxyl group
Poly(ethyleneimine) nanogels	Size-dependent property nanogel	Suicide gene hTERT –CD-TK delivered for lung cancer
Acylate group modified cholesterol bearing pullulan	Cross-linked raspberry-like assembly nanogel	Efficient interleukin-12 encapsulation and plasma levels
Poly(N-isopropylacrylamide-co-acrylamide)	*In situ* gelatinised thermo-sensitive nanogel	Drug loading capacity, bovine serum albumin
Glycol chitosan grafted with 3-diethylaminopropyl groups	pH-responsive	Doxorubicin uptake accelerated
Acetylated hylauronic acid	Specific targeting nanogel	Doxorubicin loaded nanogel
Pluronic poly(ethyleneimine)	Temperature responsive and volume transition nanogels	Thermo responsive endosomal rupture by nanogel and drug release
